# Diagnostic Strategies for Recurrent Cervical Cancer: A Cohort Study

**DOI:** 10.3389/fonc.2020.591253

**Published:** 2020-12-07

**Authors:** Xiaopei Chao, Junning Fan, Xiaochen Song, Yan You, Huanwen Wu, Ming Wu, Lei Li

**Affiliations:** ^1^Department of Obstetrics and Gynecology, Peking Union Medical College Hospital, Beijing, China; ^2^Department of Epidemiology and Biostatistics, School of Public Health, Peking University Health Science Center, Beijing, China; ^3^Department of Pathology, Peking Union Medical College Hospital, Beijing, China

**Keywords:** uterine cervical cancer, recurrence, diagnosis, symptom, imaging, physical examination

## Abstract

**Objective:**

The effectiveness of various strategies for the post-treatment monitoring of cervical cancer is unclear. This pilot study was conducted to explore recurrence patterns in and diagnostic strategies for patients with uterine cervical cancer who were meticulously followed using a customized monitoring plan.

**Methods:**

The epidemiological and clinical data of patients with recurrent cervical cancer treated from March 2012 to April 2018 at a tertiary teaching hospital were retrospectively collected. The diagnostic methods and their reliability were compared across patients with various clinicopathological characteristics and were associated with survival outcomes.

**Results:**

Two hundred sixty-four patients with recurrent cervical cancer were included in the study, among which recurrence occurred in the first three years after the last primary treatment in 214 patients (81.06%). Half of the recurrence events (50.76%) occurred only within the pelvic cavity, and most lesions (78.41%) were multiple in nature. Among all recurrent cases, approximately half were diagnosed based on clinical manifestations (n=117, 44.32%), followed by imaging examinations (n=76, 28.79%), serum tumor markers (n=34, 12.88%), physical examinations (n=33, 12.50%) and cervical cytology with or without high-risk human papillomavirus (hrHPV) testing (n=4, 1.52%). The reliability of the diagnostic methods was affected by the stage (*p*<0.001), primary treatment regimen (*p*=0.001), disease-free survival (*p*=0.022), recurrence site (*p*=0.002) and number of recurrence sites (*p*=0.001). Primary imaging methods (sonography and chest X-ray) were not inferior to secondary imaging methods (computed tomography, magnetic resonance imaging and positron emission tomography-computed tomography) in the detection of recurrence. The chest X-ray examination only detected three cases (1.14%) of recurrence. Patients assessed with various diagnostic strategies had similar progression-free and overall survival outcomes.

**Conclusions:**

A meticulous evaluation of clinical manifestations might allow recurrence to be discovered in a timely manner in most patients with cervical cancer. Specific diagnostic methods for revealing recurrence were not associated with the survival outcomes.

## Introduction

Uterine cervical cancer is one of most common causes of female cancer-related death among women worldwide (1). According to a conservative estimate, in 2015, 98,900 and 30,500 cases of incident cervical cancer and related mortality, respectively, occurred in China (2), accounting for one-fifth of the total number of new cases of cervical cancer worldwide (3). After standard treatment, the recurrence rates of International Federation of Gynecology and Obstetrics (FIGO) stage IB-IIA and IIB-IVA cervical cancer are 11% to 22% and 28% to 64%, respectively (4). Some studies have even reported a recurrence rate in patients with advanced cervical cancer of as high as 70% (5, 6). The treatment of recurrent cervical cancer remains challenging, and the prognosis of patients with recurrent cervical cancer still remains poor, with a 5-year overall survival (OS) rate of less than 5%, despite intensive therapy (7). Surveillance will provide benefits for patients with locally recurrent disease who are able to receive potentially curative treatment (8). The aim of surveillance is to detect relapse at a stage when salvage treatment has the best chance of being effective and to monitor and treat treatment-related toxicity after the last primary treatment, since approximately 50% and 75% of cervical cancer recurrence cases occur within the first one and two years after primary treatment, respectively (9, 10).

However, research examining the most effective strategies for surveillance after patients have achieved a complete response is lacking. The follow-up duration and schedules differ among countries and institutions. Controversy still exists regarding the conventional monitoring approaches of cytological evaluation and various imaging methods. According to the recommendations of Society of Gynecologic Oncology, counseling patients about signs and symptoms remains an important component of survivorship care for patients with cervical cancer, while the reduction in unnecessary cytology and colposcopic evaluations may provide significant cost-savings while maintaining quality of care in these patients (8). Currently, some authors have suggested that little support exists for surveillance chest X-ray, and it can be omitted (11, 12). However, these recommendations require further confirmation and validation in cohorts with larger sample size. The role of imaging evaluations in detecting recurrence is also controversial (13, 14). The survival benefits obtained from various diagnostic methods are still unknown (8).

This study explored the recurrence sites, diagnostic strategies, and potential relevant risk factors for patients with recurrent cervical cancer who were treated at a tertiary teaching hospital using a customized and stringently performed follow-up protocol.

## Methods

### Ethical Approval

The Institutional Review Board of the study center approved the study (No. ZS-1427). All patients or their representatives provided written informed consent before participation in the study. The registration number is NCT03291236 (*clinicaltrials.gov*, registered on September 27, 2017). All procedures described in the study involving human participants were performed in accordance with the ethical standards of the institutional and National Research Committee and with the 1964 *Declaration of Helsinki* and its later amendments or comparable ethical standards.

### Study Design

This retrospective study was conducted at a tertiary teaching hospital. Detailed epidemiological, clinical, pathological and follow-up data were collected by medical staff. The primary objective of the study was to provide a landscape of recurrence sites and relevant diagnostic methods in patients with cervical cancer. The secondary objective was to compare the efficiency of different diagnostic methods in detecting recurrence.

### Study Population

The study population consists of recurrent cervical cancer patients. All patients who treated for uterine cervical cancer at the study center from March 2012 to April 2018 were searched and reviewed. The patients’ medical records were deliberately reviewed to identify cases of diagnosed and treated recurrent cervical cancer. For patients without survival information, an email and/or telephone interview was performed to confirm the disease status and relevant information. The inclusion criteria consisted of the following (1): confirmed recurrence during the study period (2); histopathology of squamous cell carcinoma (SCC), endocervical adenocarcinoma, or adenosquamous carcinoma; and (3) acceptance of primary treatment and customized follow-up at the study center. Patients who did not meet the inclusion criteria, were lost to follow-up after primary treatment or did not experience recurrence were excluded.

### Interventions

#### Evaluation of Primary Tumors

The clinical and pathological characteristics of the primary tumors in the patients were collected from medical records and supplemented by interviews with the patients and/or their family members. These data consist of age at diagnosis, stage according to the FIGO 2009 staging system (15), histological subtype and differentiation, primary treatment and adjuvant therapy. According to the FIGO stage, the patients were further categorized as having early-stage disease (stage IA1 to IB1), locally advanced disease (stage IB2 to IIB), and advanced disease (stage IIIB to IV). The histological subtype and differentiation were reviewed and confirmed by two independent pathologists (HW and YY). If a patient accepted a curative treatment protocol (such as radical radiotherapy/concurrent chemoradiotherapy (CCRT) or radical hysterectomy), the treatment was defined as primary treatment. Adjuvant therapy consisted of therapeutic entities administered before and/or after the primary treatment, including chemotherapy, radiotherapy or CCRT. Radiotherapy/CCRT became an adjuvant therapy only after radical hysterectomy.

#### Follow-Up Protocols and Diagnosis of Recurrence

All patients treated at the study center accepted a customized follow-up protocol. The protocol followed current international guidelines (14–16) and studies (16–18) and was modified according to the native healthcare culture and hospital policies. Within the first year after the last treatment, the patient visited an outpatient clinic every 3 months and was interviewed, and complaints about symptoms were analyzed. A comprehensive physical examination, cervical/vaginal cytology testing with or without high-risk human papillomavirus (hrHPV) testing, a serum biomarker analysis (CA-125 for all patients and SCCAg for patients with SCC), abdominal and pelvic sonography and chest X-ray imaging were also performed. For the next year, the outpatient visit frequency was changed to every 4 months if no abnormal findings appeared. In the third to fifth years, the visit frequency was changed to every 6 months, and for the subsequent period, it was reduced to every 12 months. In addition, every 12 months, a secondary imaging assessment, including computed tomography (CT), magnetic resonance imaging (MRI) or positron emission tomography-computed tomography (PET-CT), was performed according to the preference of the patient and the potential necessity for disease evaluation. hrHPV genotyping is not compulsory at the study center and was not included in the analysis due to its uncertainty in detecting cancer recurrence, according to our experience and relevant reports (19).

The diagnosis of recurrence was based on patient complaints of symptoms, a physical examination, imaging examinations, or serum biomarker analysis. In this study, if one patient was diagnosed with recurrence using one method, the method was considered the first index assessment of recurrence. For example, if one patient complained of vaginal bleeding before screening or the symptom was confirmed by a physical examination or abnormal findings on cytology or imaging, the symptom was defined as the “diagnostic method”. In the present study, we confirmed recurrence by conducting a pathological review and/or secondary imaging evaluations. Obviously, not all recurrence cases were pathologically confirmed. A serum biomarker analysis, cytology, physical examination, complaints of symptoms and primary imaging methods (sonography and/or chest X-ray) were not utilized as methods for the confirmation of recurrence.

A detailed description of recurrence sites was provided for the involved organs, except for cases of recurrence with an unspecific location. The recurrence sites were further categorized as within and/or beyond the pelvic cavity. Based on the surgical and/or imaging findings, the number of recurrent lesions was categorized as solitary (continuous nodule or mass) and multiple (separate lesions), although a solitary lesion might involve multiple adjacent organs.

#### Utilization of Imaging Methods

In this study, transvaginal sonography (TVS), abdominal ultrasound and chest X-ray examinations were categorized as primary imaging methods due to their low cost, feasibility and frequent utilization. CT, MRI, and PET-CT were categorized as secondary imaging methods due to their high cost and intricate and subtle interpretations. As described above, a secondary imaging method was performed every 12 months or longer at our center if no abnormal findings were obtained during the routine follow-up protocol.

#### Description of Symptoms

The symptoms were all provided by the patients. A detailed description of the symptoms is provided in **Supplementary Table 1**.

### Statistics

Comparisons of continuous variables were conducted with parametric methods if the assumption of a normal distribution was confirmed. Nonnormally distributed variables and categorical data were compared using nonparametric tests. A logistic regression model was constructed to determine the potential risk factors for recurrence beyond the pelvic cavity using clinical and epidemiological parameters. Unless indicated otherwise, all analyses were performed with a two-sided significance level of 0.05 using Statistical Product and Service Solutions (SPSS) Statistics 20.0 software (IBM Corporation, Armonk, NY, USA).

## Results

### Patient Characteristics and Recurrence Sites

**Figure 1:**
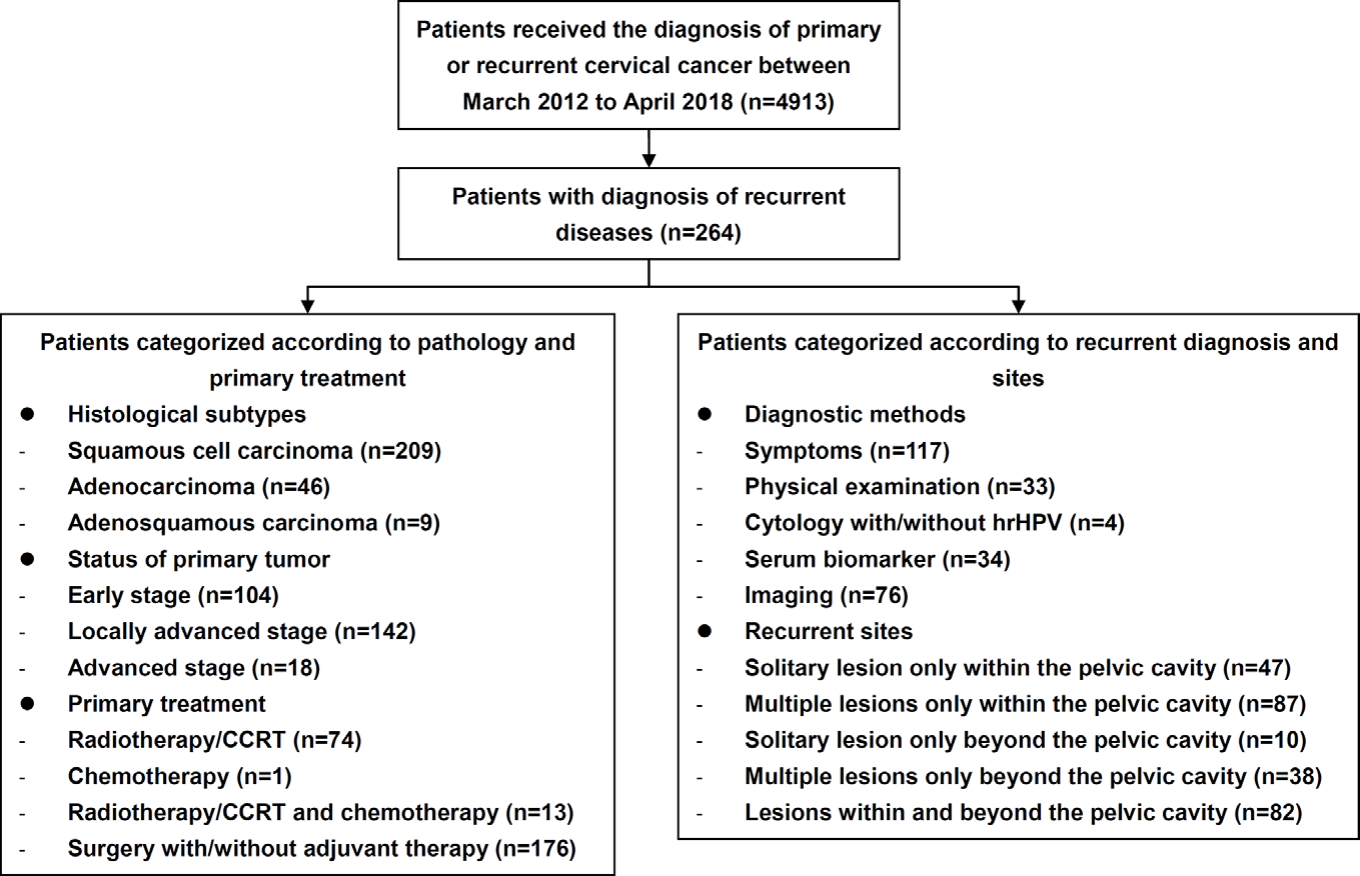
Flow chart of this study. CCRT, concurrent chemoradiotherapy.

The study flow diagram is shown in **Figure 1**. During the study period, 264 patients with recurrent cervical cancer were enrolled at the study center (**Table 1**), who were treated for their primary diseases at our center and accepted a meticulous surveillance protocol since then. Among recurrent cases, 163 (61.74%) had recurrent evidences of histopathology. The mean age was 48.69 ± 9.78 years, and the median disease-free survival (DFS) since the primary treatment was 26.85 months (3-250). Two hundred nine (79.17%), 46 (17.42%), and nine (3.41%) cases of SCC, endocervical adenocarcinoma, and adenosquamous carcinoma, respectively, were identified. One hundred four (39.39%), 142 (53.79%), and 18 (6.82%) cases of early-stage disease, locally advanced cervical cancer (LACC) and advanced disease, respectively, were identified. Regarding the initial treatment, 176 (66.67%) and 74 (28.03%) patients underwent surgical treatment and CCRT or radiotherapy, respectively. Only one patient (0.38%) underwent chemotherapy as the initial treatment. Among those 176 patients who underwent surgery as the initial treatment, abdominal and minimally invasive surgeries accounted for 59.09% (n=104) and 40.91% (n=72), respectively, of treatments.

**Table 1:** Demographic and clinicopathological characteristics of all patients.

	All patients (n = 264)
Age (years), mean ± SD)	48.69 ± 9.78
FIGO 2009 staging, n (%)	
I	142 (53.79)
II	104 (39.39)
III	13 (4.92)
IV	5 (1.89)
Staging categories, n (%)	
Early	104 (39.39)
Locally advanced	142 (53.79)
Advanced	18 (6.82)
Histological subtypes, n (%)	
SCC	209 (79.17)
ADC	46 (17.42)
Adenosquamous carcinoma	9 (3.41)
Histological differentiation, n (%)	
Grade 1	20 (12.35)
Grade 2	79 (48.77)
Grade 3	63 (38.89)
Primary treatment regimens, n (%)	
Only chemotherapy	1 (0.38)
Only radiotherapy or CCRT	74 (28.03)
Radiotherapy or CCRT plus chemotherapy	13 (4.92)
Surgery with/without adjuvant therapy	176 (66.67)
DFS (months), median (range)	16.87 (3.1–249.6)
Recurrent sites, n (%)	
Only within pelvic cavity	134 (50.76)
Only beyond pelvic cavity	48 (18.18)
Both within and beyond pelvic cavity	82 (31.06)
Number of recurrent sites, n (%)	
Solitary	57 (21.59)
Multiple	207 (78.41)
Diagnostic regimens, n (%)	
Symptoms	117 (44.32)
Physical examination	33 (12.50)
Cervical cytology with/without hrHPV	4 (1.52)
Serum biomarker	34 (12.88)
Imaging	76 (28.79)
Pathological evidences of recurrence, n (%)	
No	101 (38.26)
Yes	163 (61.74)

ADC, adenocarcinoma; CCRT, concurrent chemoradiotherapy; DFS, disease-free survival; FIGO, the International Federation of Gynecology and Obstetrics; hrHPV, high-risk human papillomavirus; SCC, squamous cell carcinoma; SD, standard deviation.

**Figure 2:**
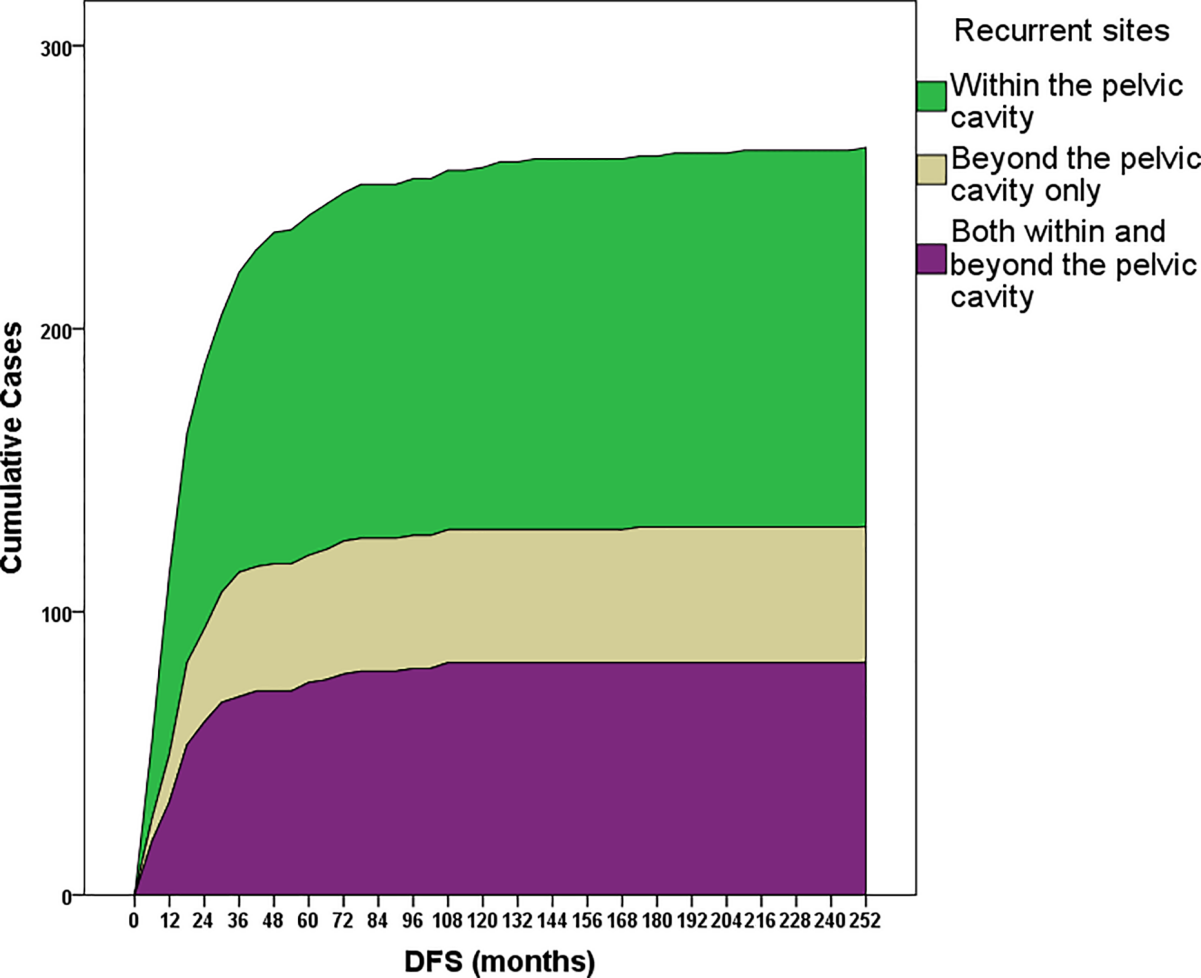
Cumulative recurrence cases within subgroups stratified according to the recurrence site: only within or only beyond the pelvic cavity or both within and beyond the pelvic cavity. Most recurrence cases (81%) occurred within the first 3 years after the last date of primary treatment.

Overall, 35.23% (n=93), 66.67% (n=176), 81.06% (n=214), and 87.88% (n=232) of recurrence cases occurred within the first one, two, three and four years since the last treatment, respectively (**Figure 2**). Recurrence within, beyond, and both within and beyond the pelvic cavity during the first three years occurred in 76.86% (103/134), 87.50% (42/48), and 84.15% (69/82) of patients, respectively (*p*=0.188). However, 90.00% (63/70) and 69.52% (73/105) of patients treated with minimally invasive surgery and open surgery, respectively, experienced recurrence within the first three years (*p=*0.001), although these two surgical groups were similar in terms of the distribution of early-stage disease versus LACC (*p=*0.071), radical hysterectomy (*p=*0.251), age at diagnosis (*p=*0.467), and pre- or postsurgical adjuvant therapy (*p=*0.107 and 0.346, respectively). Additionally, patients treated with minimally invasive surgery experienced recurrence earlier than patients treated with open surgery (median DFS: 13.9 [range, 3.3–65.5] vs 22.4 [3.1–174.5], *p*<0.001).

**Figure 3:**
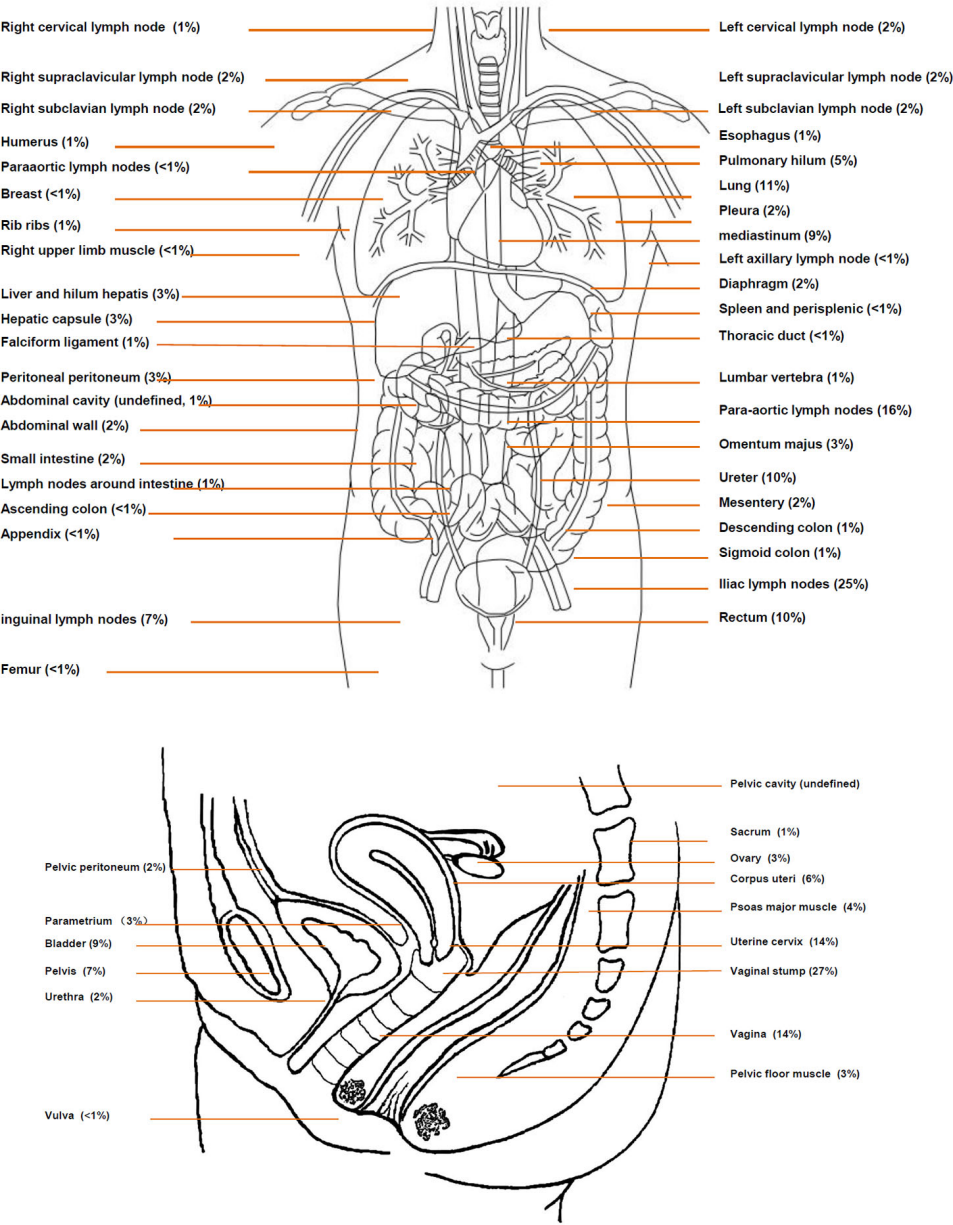
Definite distribution of recurrence sites. The percentage shows the proportion of recurrence sites in the entire population, and thus the total rounded number is greater than 100%. As half of recurrence cases occurred in the pelvic cavity, an amplifying figure is provided to describe the details of recurrence in the pelvic cavity.

As shown in **Table 1**, approximately half of recurrence cases occurred only within the pelvic cavity (50.76%). Recurrence only beyond the pelvic cavity or both within and beyond the pelvic cavity occurred in 18.18% and 31.06% of patients, respectively. Most lesions (78.41%) were multiple, and only 21.59% of lesions were single lesions. The details of the recurrence sites are shown in **Figure 3**. Among all cases, the most frequent recurrence sites (>10%) were the vaginal stump (n=70, 26.51%), lymph nodes of iliac blood vessels (n=65, 24.62%), para-aortic lymph nodes (n=42, 15.91%), vagina (n=38, 14.39%), uterine cervix (n=36, 13.64%), lungs (n=30, 11.36%), and ureters (n=27, 10.23%). Among patients diagnosed with solitary lesions within the pelvic cavity (n=47), recurrence was most commonly observed in the vaginal stump (n=33, 70.21%), followed by the vagina (n=4, 8.51%), ovaries (n=2, 8.51%), and rectum (n=2, 4.26%).

### Risk Factors for Recurrence Beyond the Pelvic Cavity

The potential risk factors contributing to recurrence beyond the pelvic cavity are listed in **Table 2**. The tumor stage (*p*=0.004), primary treatment regimen (*p*=0.005), surgical protocol (radical hysterectomy or not, *p*=0.042), and adjuvant therapy after the primary treatment regimen (*p*=0.012) showed statistical significance. Among all patients treated surgically (n=176) and among patients with early-stage disease (n=104), a significant difference in distant recurrence was not observed between patients who underwent open and minimally invasive surgery (*p*=0.908 and 0.651, respectively). Based on the tumor stage, primary treatment regimen, surgical protocol and adjuvant therapy, the logistic regression analysis revealed that adjuvant therapy after the initial treatment was an independent risk factor for distant recurrence. Patients who were not treated with adjuvant chemotherapy had a hazard ratio (OR) of distant recurrence of 2.5 (95% confidence interval [95% CI): 1.1–5.9, *p*=0.038) compared with patients who were treated with adjuvant chemotherapy. The effect of chemotherapy on distant recurrence was also evident in patients with early-stage disease (HR: 7.8, 95% CI: 1.6–37.4, *p*=0.010), but the difference was not statistically significant in patients with LACC (HR: 1.2, 95%: CI 0.4–3.4, *p*=0.775).

**Table 2:** Potential risk factors predicting recurrence beyond the pelvic cavity.

	Only within pelvic cavity (n = 134)	Beyond pelvic cavity (n = 130)	*p*
Age (years), mean ± SD	48.95 ± 10.51	48.42 ± 9.41	0.664
FIGO 2009 staging, n (%)			**0.002**
I	85 (63.43)	57 (43.85)	
II	46 (34.33)	58 (44.62)	
III	2 (1.49)	11 (8.46)	
IV	1 (0.75)	4 (3.08)	
Staging categories, n (%)			**0.004**
Early	61 (45.52)	43 (33.08)	
Locally advanced	70 (52.24)	72 (55.38)	
Advanced	3 (2.24)	15 (11.54)	
Histological subtypes, n (%)			0.879
SCC	107 (79.85)	102 (78.46)	
ADC	22 (16.42)	24 (18.46)	
Adenosquamous carcinoma	5 (3.73)	4 (3.08)	
Histological differentiation, n (%)			0.719
Grade 1	9 (11.11)	11 (13.58)	
Grade 2	42 (51.85)	37 (45.68)	
Grade 3	30 (37.04)	33 (40.74)	
Primary treatment regimens, n (%)			**0.005**
Only chemotherapy	1 (0.75)	0 (0)	
Only radiotherapy or CCRT	27 (20.15)	47 (36.15)	
Radiotherapy or CCRT plus chemotherapy	4 (2.99)	9 (6.92)	
Surgery with/without adjuvant therapy	102 (76.12)	74 (56.92)	
Surgical routes, n (%)			0.908
Laparoscopy	41 (40.20)	29 (39.73)	
Open surgery	60 (59.41)	44 (60.27)	
Surgical protocols, n (%)			**0.042**
Radical hysterectomy	71 (68.93)	61 (82.43)	
Others	32 (31.07)	13 (17.57)	
Ovarian reservation, n (%)			0.364
No	57 (55.34)	46 (62.16)	
Yes	46 (44.66)	28 (37.84)	
Adjuvant therapy before primary treatment regimens, n (%)			0.543
No	114 (85.07)	107 (82.31)	
Yes	20 (14.93)	23 (17.69)	
Definite adjuvant therapy before primary treatment regimens, n (%)			0.050
Chemotherapy	20 (100.00)	19 (82.61)	
Chemotherapy plus radiotherapy	0 (0)	4 (17.39)	
Adjuvant therapy after primary treatment regimens, n (%)			0.340
No	87 (64.93)	77 (59.23)	
Yes	47 (35.07)	53 (40.77)	
Definite adjuvant therapy after primary treatment regimens, n (%)			**0.012**
Chemotherapy	21 (44.68)	9 (17.31)	
Radiotherapy	16 (34.04)	27 (51.92)	
Chemotherapy plus radiotherapy	10 (21.28)	16 (30.77)	
DFS after primary treatment (months), median (range)	15.92 (3.1–249.6)	17.18 (4.3–174.5)	0.191

ADC, adenocarcinoma; CCRT, concurrent chemoradiotherapy; DFS, disease-free survival; FIGO, the International Federation of Gynecology and Obstetrics; SCC, squamous cell carcinoma; SD, standard deviation.

Bold values show the p values <l0.05.

### Efficacy of Diagnostic Methods for Detecting Recurrence

Approximately half of the 264 patients with recurrent cervical cancer were diagnosed based on clinical manifestations (n=117, 44.32%), followed by imaging examinations (n=76, 28.79%), a serum tumor marker analysis (n=34, 12.88%), physical examination (n=33, 12.50%) and cervical cytology (n=4, 1.52%). For patients without evident symptoms, cytology, physical examinations, serum biomarker analysis and imaging evaluations revealed 2.72% (4/147), 22.45% (33/147), 23.13% (34/147), and 51.70% (76/147) of recurrence cases, respectively. Specifically, among patients with isolated pelvic lesions, 76.60% (36/47) were diagnosed based on clinical manifestations or on a physical examination.

The diagnostic efficacy of different methods in patient subgroups is presented in **Supplementary Table 2**. Cervical cytology was included in the physical examination category due to its limited sample size (n=4). The diagnostic method showed a significant association with the subgroups of patients stratified according to tumor stage (*p*<0.001), primary treatment regimen (*p*=0.004), DFS (*p*=0.022), and recurrence site (*p*=0.002). Patients with evident symptoms had a significantly longer DFS after treatment for primary diseases than patients identified with other diagnostic methods. Compared with clinical manifestations, the serum biomarker analysis and imaging evaluations discover more recurrence events in patients with more advanced disease, distant recurrence, and multiple lesions.

The diagnostic efficacy of the primary and secondary imaging methods is presented in **Supplementary Table 3**. Thirty-three recurrence cases were detected using primary imaging methods, including sonography for 30 cases and chest X-ray for three cases; 43 cases were detected using secondary imaging methods, consisting of CT, MRI, and PET-CT in 17, 7, and 19 cases, respectively. A significant difference in the use of secondary imaging methods was not observed between patients with early-stage disease and patients with LACC/advanced-stage disease (43.75% [14/32] versus 65.91% [29/44], *p*=0.054). Generally, the use of these two strategies did not result in significant differences in the recurrence site. Secondary imaging methods did not identify more patients with distant recurrence (**Supplementary Table 3**).

**Figure 4:**
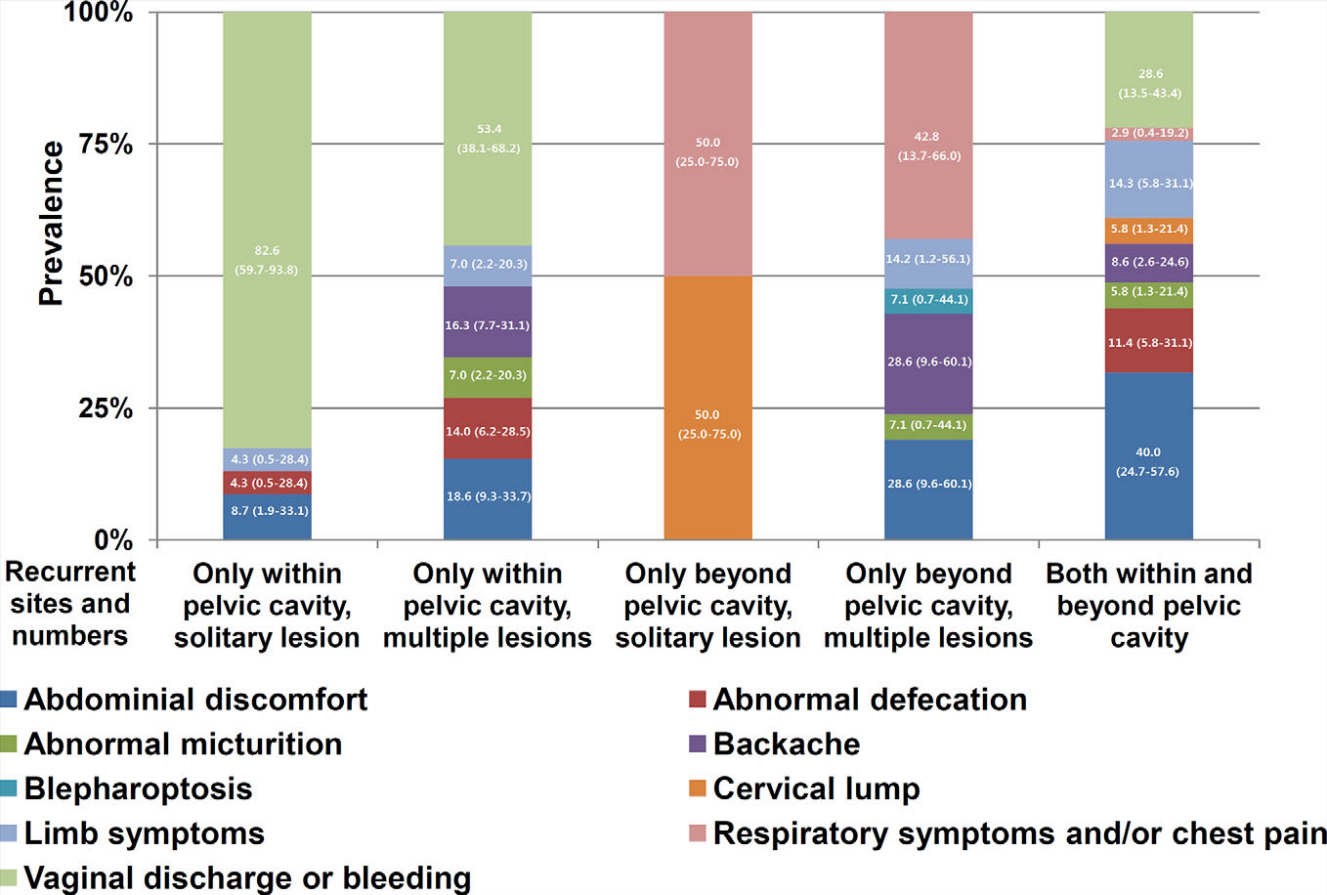
Distribution of symptoms within subgroups stratified according to the recurrence site and number of lesions (solitary or multiple). The numbers in the bars indicate percentages and their 95% confidence intervals. Abdominal pain, abdominal distention, and intestinal obstruction, as shown in **Supplementary Table 1**, were categorized as abdominal discomfort. Vulva swelling was included as a limb symptom. Respiratory symptoms and chest pain were combined as respiratory symptoms and/or chest pain. Generally, the symptoms were specific to the recurrence site and lesion number.

The associations of various symptoms with the recurrence site/number of lesions are illustrated in **Figure 4**. Due to the manifold, heterogeneous and diverse complaints, the analysis of the associations shown in **Figure 4** did not achieve sufficient power (*p* value for the linear-by-linear association=0.055). However, the symptoms still appeared to be specific to the recurrence site/number of lesions, particularly vaginal bleeding/discharge related to pelvic recurrence. Vaginal discharge or bleeding identified 82.61% and 46.00% of patients with solitary and multiple recurrent pelvic lesions, respectively. The sensitivity and specificity of vaginal bleeding/discharge for pelvic recurrence were 50.50% (51/101) and 100.00% (16/16), respectively, while the sensitivity and specificity of respiratory symptoms and/or chest pain for recurrence in the respiratory system were 53.85% (7/13) and 100.00% (104/104), respectively.

### Survival Outcomes of Patients Diagnosed Using Various Strategies

Definite survival outcomes were available for 237 patients after their treatment for recurrences. The median progression-free survival (PFS) and overall survival (OS) were 6.97 (range 0–94) and 24.03 (1.8–149.1) months, respectively. According to the Kaplan-Meier analysis, patients with recurrent cervical cancer diagnosed using various strategies had similar PFS (*p*=0.060) and OS (*p*=0.118). Compared with patients diagnosed using imaging methods or experimental testing (biomarkers or cytology), patients with clinical manifestations (symptoms or physical signs) also had similar a PFS (*p*=0.070) and OS (*p*=0.262).

## Discussion

Since no uniform follow-up protocols have been defined for patients with cervical cancer, our report on recurrence provides an interesting insight into the detection of recurrence using a customized clinical follow-up plan. These data will provide substantial knowledge for physician and patient decision-making and for the improvement of patient management by healthcare agencies.

Most cases of recurrence in our study occurred within the first three years after the last primary treatment (**Figure 2**), consistent with previous reports (11, 17, 20). Recurrence within or beyond the pelvic cavity showed similar hazards ratios. However, interestingly, patients who underwent minimally invasive surgery relapsed significantly earlier than patients who underwent open surgery, despite the similar epidemiological and clinical characteristics and surgical patterns. In our study, most (90.00%) cases of recurrence occurred within the first three years in patients who underwent minimally invasive surgery, which reduced the median DFS of these patients compared with patients who underwent open surgery. A clear explanation for this finding has not been determined, since the baseline data of all patients with primary cervical cancer are awaiting summarization and analysis. However, this finding suggests that minimally invasive surgery should be applied with caution for the treatment of cervical cancer, as suggested by a recently published randomized clinical trial (21) and an epidemiological study (22). In our study, half of the patients only experienced recurrence within the pelvic cavity, and adjuvant chemotherapy was the only independent protective factor for distant recurrence, particularly in patients with early-stage disease. Systematic chemotherapy for cervical cancer has been reported to improve survival outcomes in patients with early-stage disease (23), patients with LACC (24, 25), or in both groups (26).

The findings regarding the recurrence period confirmed the importance of meticulous monitoring during follow-up, according to the guidelines of our center and other guidelines (27, 28). Currently, most of these follow-up recommendations are based on retrospective studies and expert opinions; hence, prospective multicenter randomized trials comparing routine follow-up protocols through an open-access system would provide more substantial evidence-based knowledge about the frequency of clinical visits (10). Routine follow-up visits may delay the detection of recurrence because some patients may not present symptoms until their next routine appointment (29, 30). Therefore, the presence of symptoms or the suspicion of recurrence prompted an unscheduled evaluation in approximately 40% of patients (31, 32).

In our study, patient complaints of symptoms and a physical examination potentially served as index methods, revealing 56.82% of all recurrence cases and approximately three-fourths of solitary pelvic recurrence cases, similar to previous reports (9, 10, 31, 32). These two diagnostic methods have great advantages in terms of safety, precision, cost-effectiveness, and feasibility, and should be recommended in any situation (8, 12, 28). In our study, a physical examination revealed 12.50% (33/264) and 22.45% (33/147) of recurrence cases in the whole cohort and in asymptomatic patients, respectively, similar to the findings of previous reports (12, 33, 34). Physical examination was the main method used to detect cervical cancer relapse, and should include a thorough speculum, bimanual, and rectovaginal evaluation (8).

In the whole cohort and in patients without evident symptoms, cervical cytology revealed only 1.52% (4/264) and 2.72% (4/147) of recurrence cases, respectively. Considering the frequent clinical visits and low detection rate of cervical cancer, the necessity of cytopathology in the follow-up protocol is questioned. Surveillance with cervical cytology had previously been used to detect patients with vaginal/local recurrence (17, 35). However, the recurrence detection rate of cervical cytology ranges from 0 to 17% (11). Currently, the use of cervical cytology as a surveillance strategy for cervical cancer may be omitted from the clinical perspective or be limited to once a year for the following reasons (1): cytological evidence was rarely the only abnormality, and clinical evidence of disease was often or soon thereafter apparent (2); abnormal cytology did not always suggest cervical cancer recurrence (36) (3); normal cytology does not confirm the absence of recurrence; and (4) the rates of recurrence detected using Pap cytology remain low, which was also confirmed in our study. The discrepancy between the cytology results and disease relapse was due to the unreliable accuracy of the cytology results, which may be affected in patients who have received pelvic radiation (12, 27) or who have undergone radical hysterectomy plus pelvic lymphadenectomy for early-stage cervical cancer (37). For patients treated with primary radiation therapy, the incidence of an abnormal Pap test ranges from 6% to 34%, with atypical squamous cells of undetermined significance (ASC-US) findings accounting for most of the abnormalities (38, 39). Thus, clinicians have advocated that an evaluation of abnormal post-treatment cytology results using colposcopy should not be performed if cytology reveals changes that are less than high grade (40, 41). The cost of cytological surveillance, including colposcopy when necessary, and low rate of recurrence diagnosis may outweigh the benefit of detection. Waiving the requirement for unnecessary cytology and colposcopy evaluations may provide significant cost savings while maintaining the quality of care for these patients. Due to limited evidence for a role of hrHPV in the surveillance of cervical cancer (8), hrHPV is not currently incorporated in the follow-up strategies used at our center and in current guidelines (27).

Imaging has also been suggested for surveillance in asymptomatic patients with cervical cancer (8). In our study, the low-cost, primary imaging methods showed similar efficacy in detecting distant recurrence, suggesting the important role of these imaging evaluations in follow-up observations. Although the role of imaging is widely recognized, its use is still not standardized. Two issues remain controversial. The first is the role of a chest X-ray examination in detecting asymptomatic recurrence. Chest radiography has been recommended to be performed every 2 months within the 8 months after treatment and every 6 months thereafter (42). The detection rate of chest radiography ranges from 20% to 47% for pulmonary recurrence (11, 28). However, although successful treatment of cases of isolated pulmonary recurrence has been reported, many of these patients are not treatable (9, 43). Currently, little support exists for routine surveillance using a chest X-ray examination, and its omission has been suggested (11). In our study, the chest X-ray examination revealed 4.76% (7/147) of asymptomatic recurrence cases.

The role of secondary imaging methods in the follow-up of patients with cervical cancer remains controversial. In our study, although primary and secondary imaging methods revealed a similar number of recurrence cases (33 and 43 cases, respectively), primary imaging methods were not necessarily superior at screening patients, considering the greater application of these methods than secondary imaging methods. In contrast, in our customized follow-up protocol, more patients were diagnosed using secondary imaging methods, which were only performed yearly, highlighting the efficiency of these methods. Little is known about which secondary imaging method displays the best accuracy and cost-effectiveness. Numerous reports have highlighted the roles of CT (44), MRI (45), PET/PET-CT (46, 47), and PET-MRI (48) in the diagnosis of recurrent cervical cancer. PET has increased sensitivity and specificity (31) and the ability to detect asymptomatic recurrent disease, which is amenable to additional curative therapy (32), despite conflicting results regarding cost-effectiveness (13, 14). In 2006, the benefits of PET for detecting recurrent cervical cancer exceeded those of CT-MRI due to the ability of PET to identify extrapelvic metastases and its higher sensitivity and specificity (49). However, before high-level evidence is obtained, the application of these methods requires an individualized evaluation. New imaging techniques, such as T2-weighted imaging (T2W1) plus apparent diffusion coefficient (ADC) mapping (50, 51) and heat shock protein 70 (HSP70) mapping (52), could also be incorporated in follow-up protocols to verify their efficacy.

Although specific and sensitive biomarkers are not available for cervical cancer, an elevated CA-125 or SCCAg level remains a cause for concern and facilitated the detection of recurrence in 12.88% of patients, which is greater than the proportion discovered by cytology or chest X-ray examination, according to a previous report by Guo et al. (53). As shown in the study by Kotowicz et al. (54), SCCAg was an independent prognostic factor for DFS and OS in patients with SCC. The serum SCCAg level potentially reflects the response to treatment, and increasing antigen levels often precede the clinical detection of recurrent disease, leading to an early diagnosis (55, 56). Hence, in a follow-up protocol, serum biomarkers still play important roles. In a prospective trial, other markers, such as the neutrophil-lymphocyte ratio (57) and levels of VEGF, CYFRA 21.1, IL-6 (54), and/or new genetic/epigenetic biomarkers, could also be tested to determine their predictive efficacy.

The large cohort of patients with recurrent disease and detailed information is the strength of this study. However, several limitations must also be noted. Due to the nature of retrospective observational studies, an analysis of the cost-effectiveness of the follow-up schemes cannot be performed, as this analysis must be performed prospectively. The survival of patients with recurrence should be clarified to further validate the efficacy of the follow-up protocols (8). The potential risk factors for recurrence and relevant recurrence sites also must be specified to establish individualized protocols. A clear understanding of these issues will provide more practical and unbiased information for the efficient prevention of disease recurrence and appropriate decision-making by physicians and patients.

## Conclusions

According to a customized follow-up protocol, as high as 81.06% recurrence occurred within 3 years and approximately half of recurrent cervical cancer cases occurred within the pelvis alone. Symptoms reported by patients and a physical examination revealed more than half of the recurrence cases. More complex imaging evaluations, such as CT, MRI, and PET or PET-CT, did not identify more distant recurrence cases. Cervical cytology and an X-ray examination might be able to be omitted from the supervision system for cervical cancer due to their low detection rates. Diagnostic strategies for recurrence were not associated with subsequent survival outcomes.

## Data Availability Statement

The raw data supporting the conclusions of this article will be made available by the authors, without undue reservation.

## Ethics Statement

The studies involving human participants were reviewed and approved by Institutional Review Board of Peking Union Medical College Hospital. The patients/participants provided their written informed consent to participate in this study.

## Author Contributions

MW and LL conceived the original idea for the study, interpreted the results, performed the statistical analysis, edited the paper, and were the overall guarantors. JF interpreted the results, performed the statistical analysis, and edited the paper. XC obtained ethical approval, contributed to the preparation of the dataset, interpreted the results, and contributed to drafting the paper. MW, LL, XS, and XC contributed to the study design, interpretation of results, and commented on drafts of the paper. YY and HW conducted the pathological evaluation. All authors contributed to the article and approved the submitted version.

## Funding

This study was supported by the CAMS Innovation Fund for Medical Sciences (CIFMS-2017-I2M-1-002). The funders had no role in the study design, data collection and analysis, decision to publish, or preparation of the manuscript.

## Conflict of Interest

The authors declare that the research was conducted in the absence of any commercial or financial relationships that could be construed as a potential conflict of interest.
