# Supplementary material for: Diagnostic Strategies for Recurrent Cervical Cancer: A Cohort Study
Source: Front Oncol. 2020 Dec 7;10:591253. doi: 10.3389/fonc.2020.591253 (PMC7750634; doi:10.3389/fonc.2020.591253)
Supplement: Supplementary file 2 [file Table_2.docx]

Supplementary Table 2

The diagnostic efficacy of different methods in patient subgroups

|  | Symptoms | Physical examination* | Serum biomarker | Imaging | *p* |
| --- | --- | --- | --- | --- | --- |
| Age (years), (mean ± SD) | 48.53 ± 10.00 | 51.24 ± 10.77 | 49.82 ± 7.55 | 47.18 ± 9.68 | 0.186 |
| FIGO 2009 staging, n (%) |  |  |  |  | **0.002** |
| I | 70 (59.83) | 16 (43.24) | 11 (32.35) | 45 (59.21) |  |
| II | 44 (37.61) | 20 (54.05) | 16 (47.06) | 24 (31.58) |  |
| III | 2 (1.71) | 0 (0) | 6 (17.65) | 5 (6.58) |  |
| IV | 1 (0.85) | 1 (2.70) | 1 (2.94) | 2 (2.63) |  |
| Staging categories, n (%) |  |  |  |  | **<0.001** |
| Early | 54 (46.15) | 14 (37.84) | 4 (11.76) | 32 (42.11) |  |
| Locally advanced | 60 (51.28) | 22 (59.46) | 23 (67.65) | 37 (48.68) |  |
| Advanced | 3 (2.56) | 1 (2.70) | 7 (20.59) | 7 (9.21) |  |
| Histological subtypes, n (%) |  |  |  |  | 0.553 |
| SCC | 93 (79.49) | 32 (86.49) | 29 (85.29) | 55 (72.37) |  |
| ADC | 20 (17.09) | 5 (13.51) | 4 (11.76) | 17 (22.37) |  |
| Adenosquamous carcinoma | 4 (3.42) | 0 (0) | 1 (2.94) | 4 (5.26) |  |
| Histological differentiation, n (%) |  |  |  |  | 0.966 |
| Grade 1 | 8 (11.59) | 3 (13.64) | 1 (6.25) | 8 (14.55) |  |
| Grade 2 | 33 (47.83) | 12 (54.55) | 8 (50.00) | 26 (47.27) |  |
| Grade 3 | 28 (40.58) | 7 (31.82) | 7 (43.75) | 21 (38.18) |  |
| Primary treatment regimens, n (%)† |  |  |  |  | **0.001** |
| Only radiotherapy or CCRT | 19 (16.38) | 12 (32.43) | 19 (55.88) | 24 (31.58) |  |
| Radiotherapy or CCRT plus chemotherapy | 7 (6.03) | 1 (2.70) | 2 (5.88) | 3 (3.95) |  |
| Surgery with/without adjuvant therapy | 90 (77.59) | 24 (64.86) | 13 (38.24) | 49 (64.47) |  |
| DFS (months), median (range) | 19.23 (3.1-249.6) | 16.17 (3.2-174.5) | 16.55 (5.0-42.2) | 15.23 (4.7-126.5) | **0.022** |
| Recurrent sites, n (%) |  |  |  |  | **0.002** |
| Only within pelvic cavity | 66 (56.41) | 26 (70.27) | 7 (20.59) | 35 (46.05) |  |
| Only beyond pelvic cavity | 16 (13.68) | 5 (13.51) | 11 (32.35) | 16 (21.05) |  |
| Both within and beyond pelvic cavity | 35 (29.91) | 6 (16.22) | 16 (47.06) | 25 (32.89) |  |
| Distant recurrence, n (%) |  |  |  |  | **<0.001** |
| No | 66 (56.41) | 26 (70.27) | 7 (20.59) | 35 (46.05) |  |
| Yes | 51 (43.59) | 11 (29.73) | 27 (79.41) | 41 (53.95) |  |
| Number of recurrent sites, n (%) |  |  |  |  | **0.001** |
| Solitary | 25 (21.37) | 17 (45.95) | 5 (14.71) | 10 (13.16) |  |
| Multiple | 92 (78.63) | 20 (54.05) | 29 (85.29) | 66 (86.84) |  |

* Including cytology with/without hrHPV testing.

† One case of only chemotherapy was excluded.

Abbreviation: ADC, adenocarcinoma; CCRT, concurrent chemoradiotherapy; DFS, disease-free survival; FIGO, the International Federation of Gynecology and Obstetrics; SCC, squamous cell carcinoma; SD, standard deviation.
