# Supplementary material for: Diagnostic Strategies for Recurrent Cervical Cancer: A Cohort Study
Source: Front Oncol. 2020 Dec 7;10:591253. doi: 10.3389/fonc.2020.591253 (PMC7750634; doi:10.3389/fonc.2020.591253)
Supplement: Supplementary file 3 [file Table_3.docx]

Supplementary Table 3

Diagnostic efficiency of different imaging methods.

|  | Primary examination* (n=33) | Secondary examination† (n=43) | *p* |
| --- | --- | --- | --- |
| DFS (months), median (range) | 20.57 (5.9-126.5) | 12.33 (4.7-69.8) | 0.053 |
| Distant recurrence, n (%) |  |  | 0.193 |
| No | 18 (54.55) | 17 (39.53) |  |
| Yes | 15 (45.45) | 26 (60.47) |  |
| Recurrent sites, n (%) |  |  | 0.209 |
| Only within pelvic cavity | 18 (54.55) | 17 (39.53) |  |
| Only beyond pelvic cavity | 4 (12.12) | 12 (27.91) |  |
| Both within and beyond pelvic cavity | 11 (33.33) | 14 (32.56) |  |
| Number of recurrent sites, n (%) |  |  | 0.069 |
| Solitary | 7 (21.21) | 3 (6.98) |  |
| Multiple | 26 (78.79) | 40 (93.02) |  |
| Recurrent sites and their numbers, n (%) |  |  | 0.128 |
| Only within pelvic cavity, solitary lesion | 5 (15.15) | 2 (4.65) |  |
| Only within pelvic cavity, multiple lesions | 13 (39.39) | 15 (34.88) |  |
| Only beyond pelvic cavity, solitary lesion | 2 (6.06) | 1 (2.33) |  |
| Only beyond pelvic cavity, multiple lesions | 2 (6.06) | 11 (25.58) |  |
| Both within and beyond pelvic cavity | 11 (33.33) | 14 (32.56) |  |

* Including sonography ultrasound and X-ray.

† Including systemic CT, MRI and PET.

Abbreviation: CT, computed tomography; DFS, disease-free survival; MRI, magnetic resonance imaging; PET, positron emission tomography
